# Western boundary currents drive sun-coral (*Tubastraea* spp.) coastal invasion from oil platforms

**DOI:** 10.1038/s41598-022-09269-8

**Published:** 2022-03-28

**Authors:** Stella Correia Cesar Coelho, Douglas Francisco Marcolino Gherardi, Mainara Biazati Gouveia, Marcelo Visentini Kitahara

**Affiliations:** 1 Laboratory of Ocean and Atmosphere Studies (LOA), Earth Observation and Geoinformatics Division, National Institute for Space Research (INPE), São José dos Campos, SP Brazil; 2 Earth and Environmental Physics Department (DFTMA), Physics Institute, Ondina Campus, Federal University of Bahia (UFBA), Salvador, Brazil; 3 Institute of Marine Sciences, Federal University of São Paulo (UNIFESP), Santos, Brazil; 4 Center for Marine Biology, University of São Paulo (USP), São Paulo, Brazil; 5 Department of Zoology (Invertebrate Zoology), National Museum of Natural History, Smithsonian Institution, Washington, D.C. USA

**Keywords:** Coral reefs, Ecological modelling, Ocean sciences

## Abstract

Most marine species have a planktonic larval phase that benefit from the surface oceanic flow to enhance their dispersion potential. For invasive species, the interaction of environmentally resistant larvae with different flow regimes and artificial substrates can lead to complex larval dispersion patterns and boost geographic expansion. In the Southwest Atlantic, the invasive corals *Tubastraea* spp. (sun-coral) have been recorded biofouling on oil platforms since the late 1980s. These platforms are considered important vectors for the established populations throughout the Brazilian coast. However, we still do not know how the position of these structures relative to regional flow contribute to the natural dispersion potential of these invaders on a regional scale. Herein, we used an eddy-resolving ocean model (ROMS) and an Individual Based Model (IBM-Ichthyop) to simulate the natural dispersion patterns of sun-coral larvae from all oil platforms on Brazilian oil-producing basins, for the austral summer and winter along 6 years (2010–2015) in 90-day simulations. We found that mortality rates by advection were significantly higher during the winter (*p* = 0.001) and when sources of larvae were compared throughout this season (*p* = 1.9 × 10^–17^). The influence of two western boundary currents and persistent eddy activity contribute to the dispersal of larvae to distances up to 7000 km. The effectiveness of each oil-producing basin as vectors for the entire Brazilian coastline, measured as the percentage of larval supply, highlights the importance of the northern Ceará (59.89%) and Potiguar (87.47%) basins and the more central Camamu (44.11%) and Sergipe-Alagoas (39.20%) basins. The poleward shift of the Southern branch of the South Equatorial Current during the winter causes larvae released from the Sergipe-Alagoas and Camamu basins to enter the North Brazil Current, expanding their dispersion towards the north. The Brazil Current disperses larvae southwards, but strong mesoscale activity prevents their dispersion to the coast, especially for those released from the oil platforms on Campos and Santos basins. Within this complex hydrodynamic setting, a few source areas, like those in the Sergipe-Alagoas and Camamu basins, can potentially contribute to the spread of larvae along nearly all the Brazilian coast. Therefore, oil platforms act as possible chronic sources of sun-coral propagules to the coast, emphasizing the urgency for a more detailed set of actions to control and monitor these invasive exotic species.

## Introduction

Invasive exotic species are a major cause of marine biodiversity loss^1^. Through competitive exclusion of native populations, disruption of the food chain, and reduction of habitat complexity, they put at risk important ecosystem services, such as fisheries, aquaculture, and tourism^2,3^. For an organism to be considered an invader in an area, it must successfully pass through the stages of transport, introduction, establishment, and dispersal^4^. In the marine environment, these non-indigenous species can take advantage of different artificial structures to be introduced and to expand their invasion range^5–8^^.^

In the Southwest Atlantic, *Tubastraea coccinea* and *Tubastraea tagusensis*, popularly known as sun-corals, are the only invasive scleractinian corals, with their first record dates from the late 1980s^9^ on offshore oil platforms in the Campos Basin (Rio de Janeiro state). Native from the Indo-Pacific, these invaders have been negatively impacting benthic communities in several Brazilian marine habitats^10–13^. Currently, sun-corals are spread for more than 3500 km of the Brazilian coast, with reports on natural and artificial substrates (as oil platforms), including marine protected areas^8,14^. Despite the advances in the understanding of their reproduction and development strategies^15–18^, distribution patterns/invasion process^8,19–21^, and effectiveness of control actions^22–24^, the potential for natural dispersion of their larvae from oil platforms remains unclear on both regional and global scales.

As biofouling on oil platforms, sun-corals are already an issue in several locations in the Gulf of Mexico^25,26^, Canary Islands^27^, and Brazil^21^. Overall, these marine structures may act as vectors for the dispersion of non-indigenous propagules through larval release^28,29^. Indeed, oil platforms behave as stepping stones, connecting oceanic regions with the coastline^30,31^. Apart from several biological traits^32^, including the plankton larval duration (PLD, the time that larvae can stay in the plankton)^33^, the success of invading new coastal localities from oil platforms likely depends on the character and variance of the regional ocean flow regimes where these structures are located.

Oil platforms in the Brazilian oil-producing marine sedimentary basins are found from shallow (< 200 m) continental shelf (e.g., Ceará, Potiguar, and Sergipe-Alagoas basins) to deeper offshore sites (e.g., Campos and Santos basins). These structures are under the influence of a complex western boundary current system, which is composed of the northwards flowing North Brazil Current (NBC) and the southward flowing Brazil Current (BC)^34^, both originate from the southern branch of the South Equatorial Current (SECs)^34^ and with a strong ocean mesoscale activity^35–37^. These currents have already been presented as important drivers of the demographic connectivity of reef fish populations among marine protected areas^38,39^. However the dispersion of invasive species, such as sun-corals, from oil platforms under this complex hydrodynamic environment is yet to be determined.

Effective management planning for invasive marine species and, therefore, management actions, depend on key knowledge including larval dispersion potential, as it may favor or hinder invasions depending on the ocean surface flow. In fact, this larvae potential will largely determine if a species will be able to expand its geographical range and/or strengthen already settled invasions^40,41^. Thus, this work aims to determine how oil platforms are connected to the coast by the dispersion of sun-coral larvae under the influence of two western Atlantic boundary current systems. The performed analysis focused on the seasonal (austral summer and winter) and interannual (2010–2015) variability of larval dispersion patterns through regional biophysical simulations in the Southwest Atlantic, between 6°N–36°S and 24°W–53°W. Vulnerable sites and likely impacts of the sun-coral colonization on the Brazilian coast are pinpointed. Our results can help stakeholders in the process of strategic decision planning by indicating likely pathways of invaders dispersal to coastal habitats mediated by artificial marine structures under different flow regimes.

## Results

### Dispersion patterns and mortality rates reinforce the role of regional circulation

**Figure 1:**
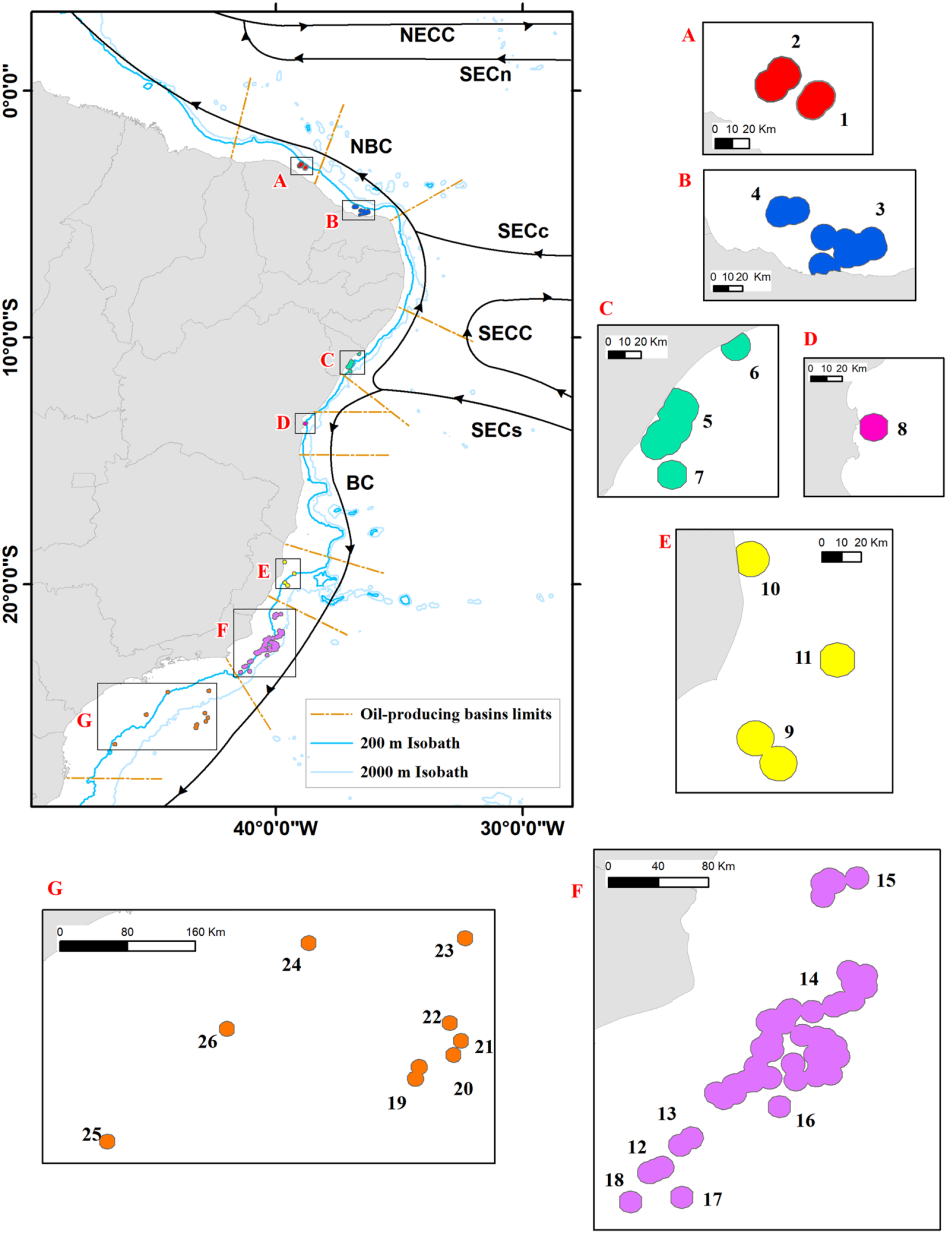
Location of the 26 source areas of sun-coral larvae distributed along the Ceará basin (A); Potiguar basin (B); Sergipe-Alagoas basin (C); Camamu basin (D); Espírito Santo basin (E); Campos basin (F) and Santos basin (G). Surface currents of the study area also represented in the map, where: NECC—North Equatorial Counter Current; SECn, SECc, SECs—South Equatorial Current ((n)orth, (c)entral and (s)outh branchs); SECC—South Equatorial Counter Current; NBC—North Brazil Current; BC—Brazil Current. Created with ArcGIS 10.6.1 (https://www.esri.com/).

Virtual trajectories of the sun-coral larvae were simulated for the austral summer (January–February–March) and winter (July–August–September) between 2010 and 2015, using hourly outputs from an eddy-resolving ocean model with a horizontal resolution of 1/12° (approximately 9.2 km) and a Lagrangian biological model (see “Data and methods”). We considered all known oil platforms located in the seven oil-producing sedimentary basins within the Brazilian Economic Exclusive Zone (Supplementary Table S2), from which we defined 26 larval release sites (source areas) (Fig. 1). Each of the 12 biophysical simulations (6 for summer and 6 for winter) was conducted as an one-release event, in which a total of 70,000 sun-coral larvae were released and allowed to drift during the 90 days pelagic larval duration (PLD), which corresponds to the maximum recorded duration for *Tubastraea coccinea* larvae in laboratory^18^.

**Figure 2:**
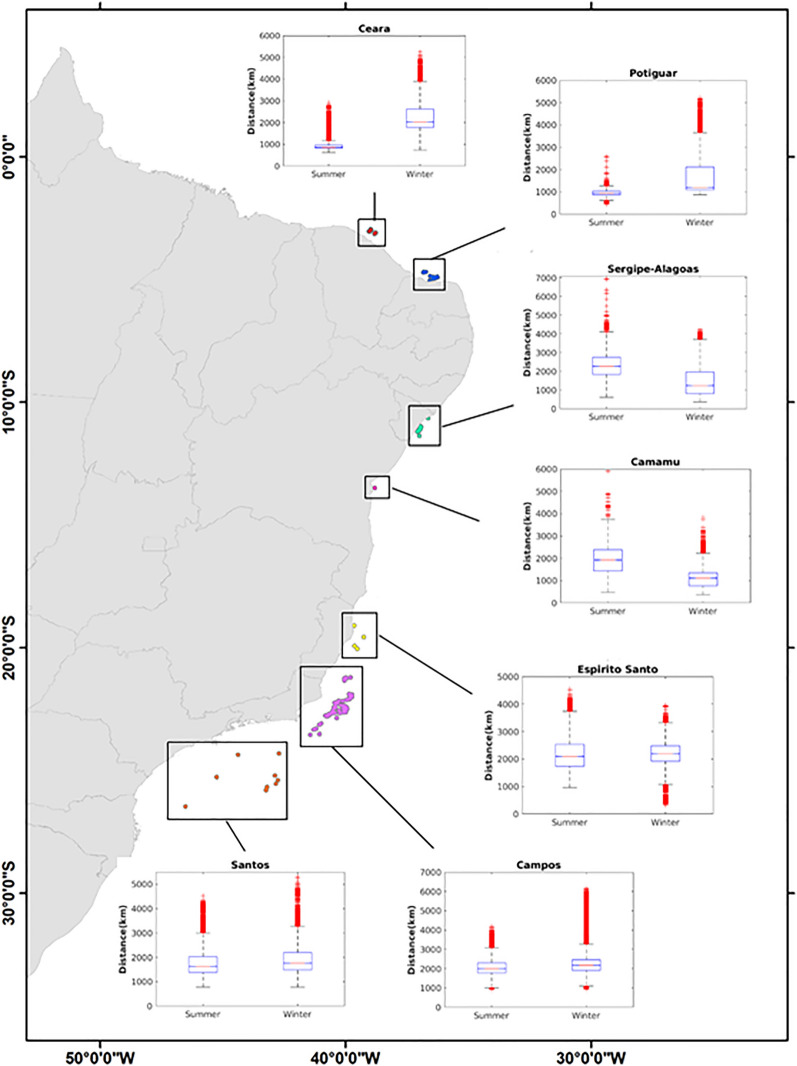
Model domain and boxplots (insert) of total distances traveled by all surviving larvae released from oil platforms (grouped by source basin) during austral summer and winter simulations. In the boxplots, the central mark indicates the median, the whiskers indicate extreme data points and red crosses are outliers. Note that the maximum values of boxplots are slightly different. Created with ArcGIS 10.6.1 (https://www.esri.com/) and Matlab R2018a (http://www.mathworks.com).

**Figure 3:**
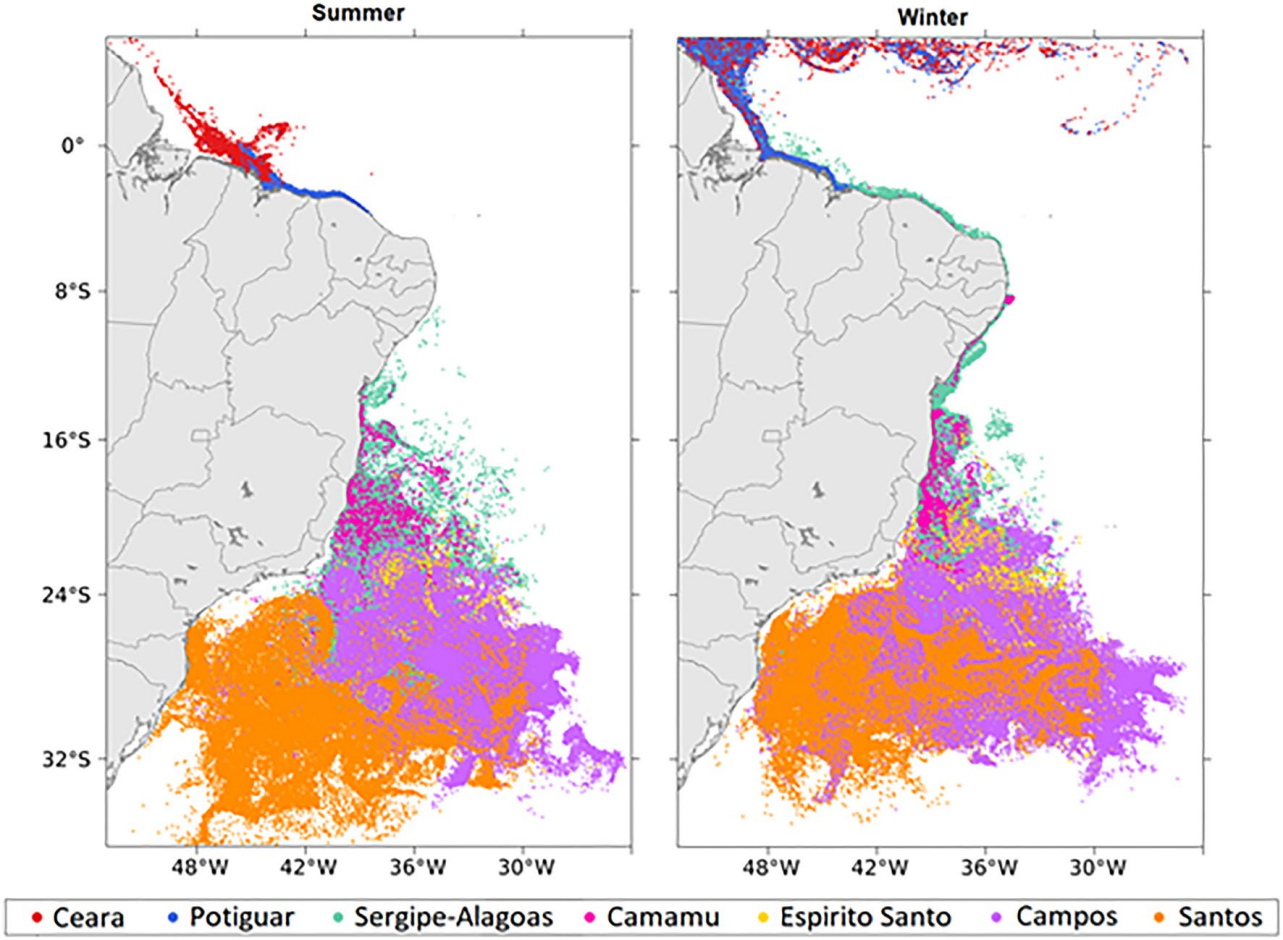
Six-year superposed final position of the surviving larvae after 90 days of simulation during the austral summer (left) and winter (right), in which each color represents the source basins. Created with Matlab R2018a (http://www.mathworks.com).

The total distance traveled by living particles, during 6 years of the experiment, shows a marked seasonality of regional circulation, especially for the northern and eastern platforms (Fig. 2). Larvae released from platforms in the northernmost Ceará and Potiguar basins travel shorter distances during the summer compared to winter. In contrast, larvae released from the Sergipe-Alagoas and Camamu platforms tend to travel longer distances during the summer compared to winter. However, there are no sharp seasonal variations in the distance traveled by the larvae released from the southeastern platforms from Espirito Santo, Campos, and Santos basins. There are also marked differences in larval trajectories inferred from their final positions, depending on where they are released and the season (Fig. 3). The narrow, coastal distribution of these positions observed for larvae released in the northern Ceará, Potiguar and the eastern Sergipe-Alagoas platforms suggests the predominance of unidirectional trajectories within coastal currents. On the other hand, more scattered final positions suggest the predominance of eddy-like and meandering trajectories further south from Camamu to Santos (Supplementary Figs. S1 and S2). Some of these larvae may travel distances exceeding 2000 km, sometimes reaching over 6000 km, but it does not mean they travel farther from their source regions as the final positions reached by most of them, in both seasons, are close to their respective origins (Fig. 3). Maximum traveled distances do not differ much among sites but, on average, larvae released from Campos and Santos basins travel longer distances than those released elsewhere as they do not display large seasonal differences as observed in larvae released further to the north.

The final positions of sun-coral larvae also reveal a seasonally-induced transport pattern with larvae released from oil platforms in the north (Ceará and Potiguar) during the summer being advected to the northwest and those released south of Sergipe-Alagoas being mostly advected to the south. Interestingly, during the winter, larvae released from the Sergipe-Alagoas and Camamu basins entered the northward flow of the North Brazil Subcurrent (NBSC) and the NBC, going as far as the Amazon River delta at the end of simulations. This suggests that these oil platforms are submitted to a possible seasonal isolation that partially segregates those located to the north (e.g.Ceará and Potiguar basins) from the rest.

Another important feature of the wintertime advection of larvae released from the northern Ceará and Potiguar oil platforms is the possibility of entering the North Equatorial Counter Current (NECC) (see Supplementary Fig. S3) increasing the chance of sun-coral larvae to cross the Amazon River delta towards the Caribbean Sea and the east Equatorial Atlantic. Although less frequent, the latter could lead to the transport of sun-coral larvae to the Marine Natural Monument of São Pedro and São Paulo Archipelago (00°53′ N, 029°16′ W), an open ocean pristine marine protected area. The larger number of oil platforms (and release sites) found to the south, especially in the Campos and Santos basins, does not seem to enhance the potential for dispersion of sun-coral towards the coast, since their endpoints are mostly located offshore (Fig. 3). Although these larvae are capable of traveling considerable distances (up to 6000 km, see Fig. 2), their trajectories tend to assume a circular and restrained pattern. These trajectories may extend as far to the east as 26° W, seen in all the simulated years for both seasons (Supplementary Fig. S4). However, the chances of these larvae to find their way to the southeast Brazil coast should not be overlooked as they approach it from the south between 24° and 32° S (Fig. 3).

**Figure 4:**
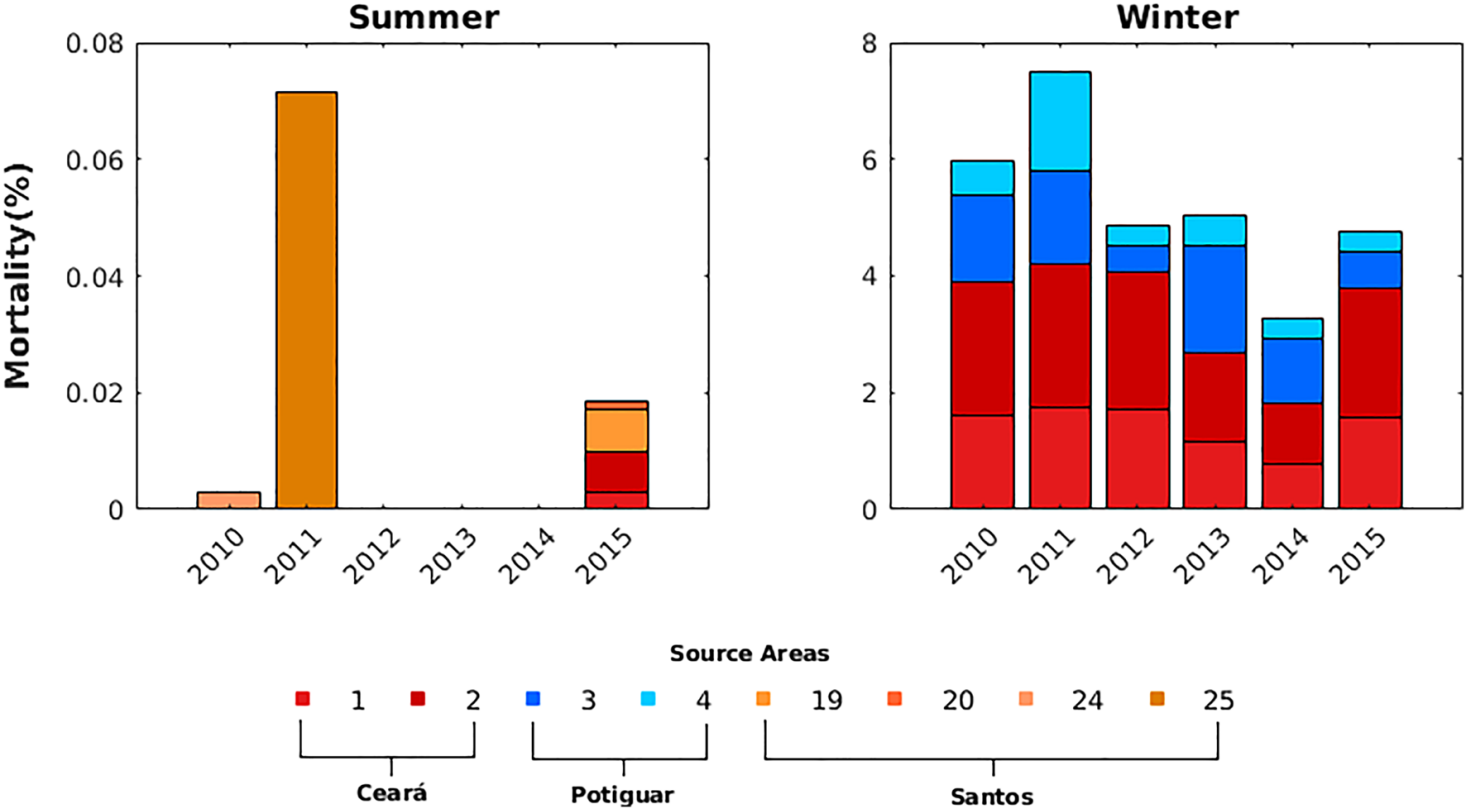
Mortality rates caused by advection for austral summer (left) and winter (right) computed for each source area between 2010 and 2015. Source areas that experienced no mortality are not represented in the summer chart. In the winter chart it is only represented source areas with mortality rates > 0.03%. Created with Matlab R2018a (http://www.mathworks.com).

The high dispersal potential of larvae released during winter from oil platforms located in the Ceará and Potiguar basins is reflected in the mortality rates by advection (Fig. 4), which occurs when larvae reach the open boundaries of the model domain. Larvae released from the Sergipe-Alagoas basin during the winter also had mortality by advection when entered the northwestward flows of the NBSC and the NBC, but at an extremely low rate (> 0.03%). In fact, these mortality rates were significantly higher during the winter compared to summer (Mann–Whitney test, *p* = 0.001), but interannual variability was not significant (Kruskal–Wallis test, *p* = 0.856). Mortality by advection during the winter was also significantly different (Kruskal–Wallis test, *p* = 1.9 × 10^–17^) when sources of larvae were compared (Fig. 4), with differences concentrated in the Ceará and Potiguar basins. Although summertime mortality by advection was observed in Ceará and Santos basins, the difference in the mortality rate between source areas was not significant (*p* = 0.7109). Advection to the outside of the model domain was the only cause of mortality recorded in our simulations as the broad temperature tolerance of these species resulted in no mortality from lethal temperatures during the experiments. Indeed, there are no known records of maximum lethal sea surface temperature (SST) value for *Tubastraea* spp. larvae, and we have, therefore, considered only the minimum lethal SST of ≤ 12.5 °C^42^ (see Table 1).

**Table 1:** Configuration of the biophysical parameters used to feed the Ichthyop simulations.

Variable	Value/attribute
Total number of released particles	70,000
Number of source areas	26
Depth of larvae releases	1–30 m
Larvae density	1016 g cm^−3^^97^
Pelagic larvae duration	90 days^18^
Lethal temperature	≤ 12.5 °C^42^
Coastline behavior	Bouncing (particles remain do not die after touching the coastline)
Advection method	Runge–Kutta 4
Turbulent dissipation rate	1 × 10^−9^
Hydrodynamic dataset	ROMS 3D/Rutgers

**Figure 5:**
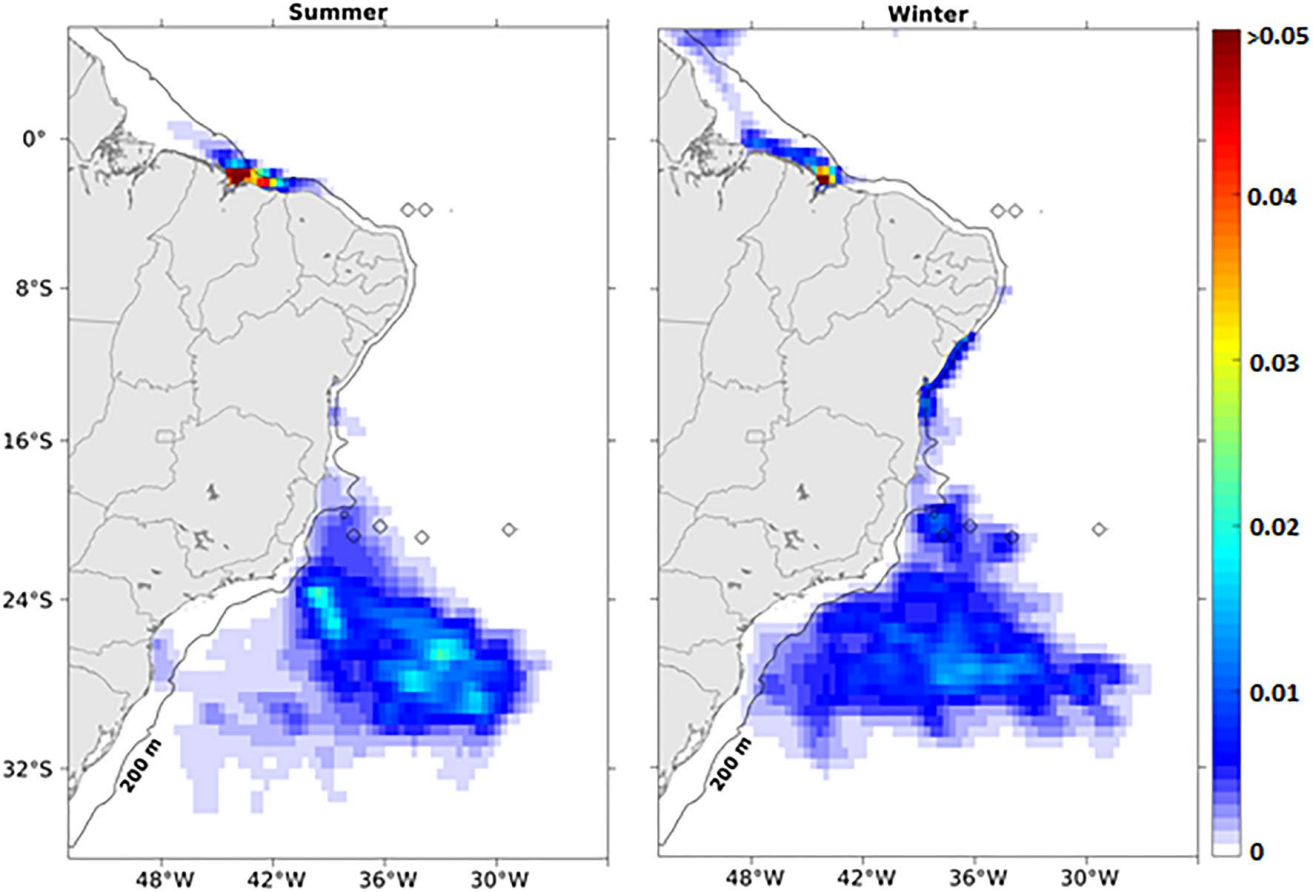
Kernel density estimation for the surviving larvae released from all the 26 source areas during the 90 days of austral summer (left) and winter (right) simulations. The empty circles on the maps are seamounts shallower than 200 m. Created with Matlab R2018a (http://www.mathworks.com).

The tendency for seasonal isolation of the northern Brazilian coast during the summer described above is also evident in the density distribution of sun-coral larvae released from oil platforms in the Southwest Tropical Atlantic (Fig. 5). There was a slightly higher density of larvae concentrated along the coast during this period, contrasting with a wider distribution of larvae within their PLD (90 days) towards the northwest during the winter. The summer larval retention on the coast reflects the predominance of inshore trajectories traveled by larvae released from Ceará and Potiguar basins in all years (Supplementary Fig. S2). Thus, such coastal retention increases the probability of the occurrence of sun-coral larvae along the coast between the oil platforms and the Amazon River delta. A wider density kernel occurs between 20 and 30°S, offshore of the 200 m isobath, that includes source areas of Campos and Santos basins, responsible for 54.8% and 15.5% of the total larvae released, respectively. In these basins, oil platforms are located in deeper waters (> 200 m) under the influence of the southward-flowing BC. Although seasonal changes of larvae density are less marked in this eddy-dominated flow region, including the Cabo Frio upwelling (23°S), larvae tend to remain offshore and more concentrated in the summer than during winter.

### Oil platform location determines their potential as vectors for sun-coral coastal invasion

**Figure 6:**
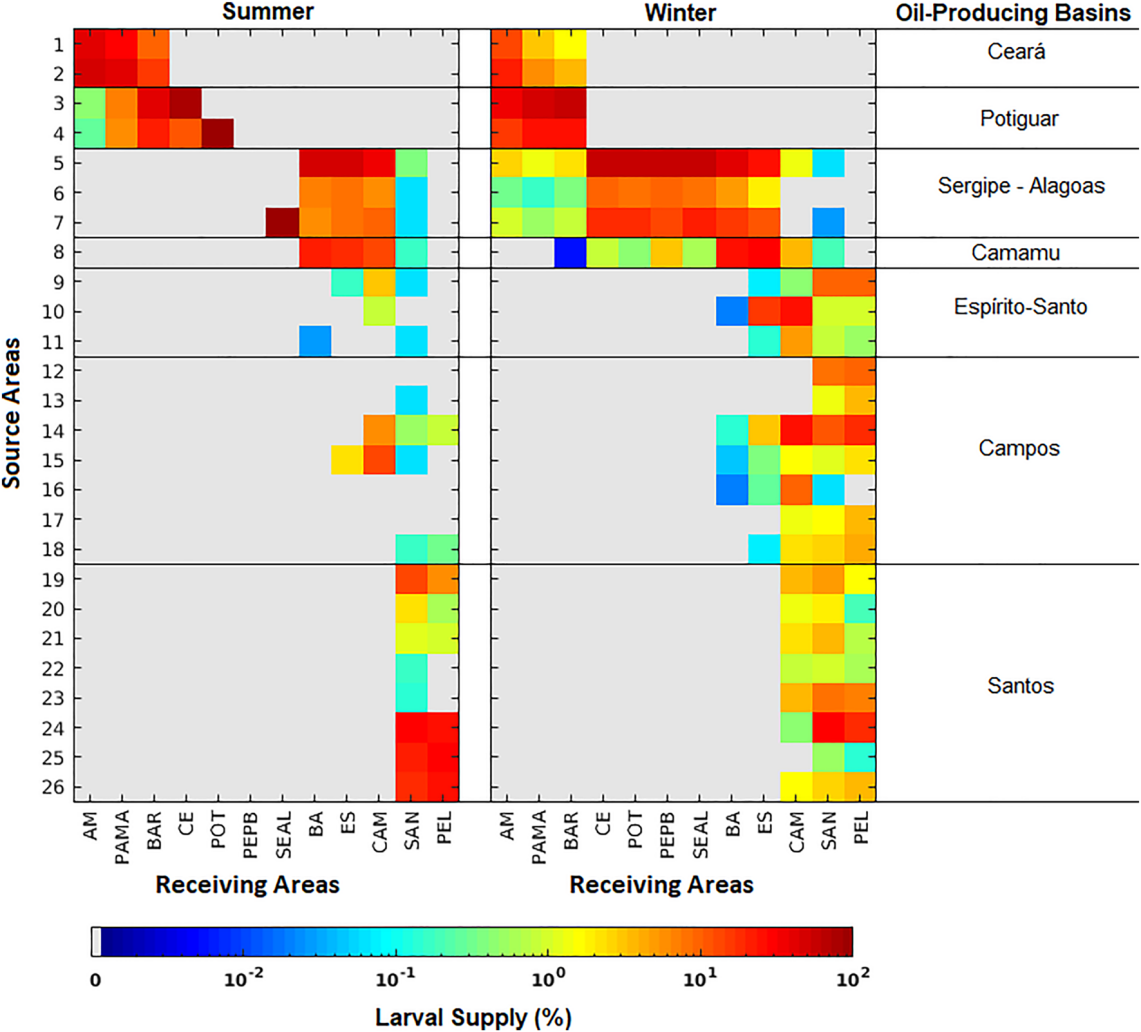
Connectivity matrix representing the relative importance of oil platforms as source areas (y-axis) of sun-coral larvae to the receiving areas (x-axis). Receiving areas are related to its location on the Brazilian coastline and abbreviated as: Amazonas (AM), Pará-Maranhão (PAMA), BAR (Barreirinhas), Ceará (CE), Potiguar (POT), Pernambuco-Paraíba (PEPB), Sergipe-Alagoas (SEAL), Bahia (BA), Espírito Santo (ES), Campos (CAM), Santos (SAN) and Pelotas (PEL). See Fig. 1 for source areas location and Fig. 8 for receiving areas location. Created with Matlab R2018a (http://www.mathworks.com).

**Figure 7:**
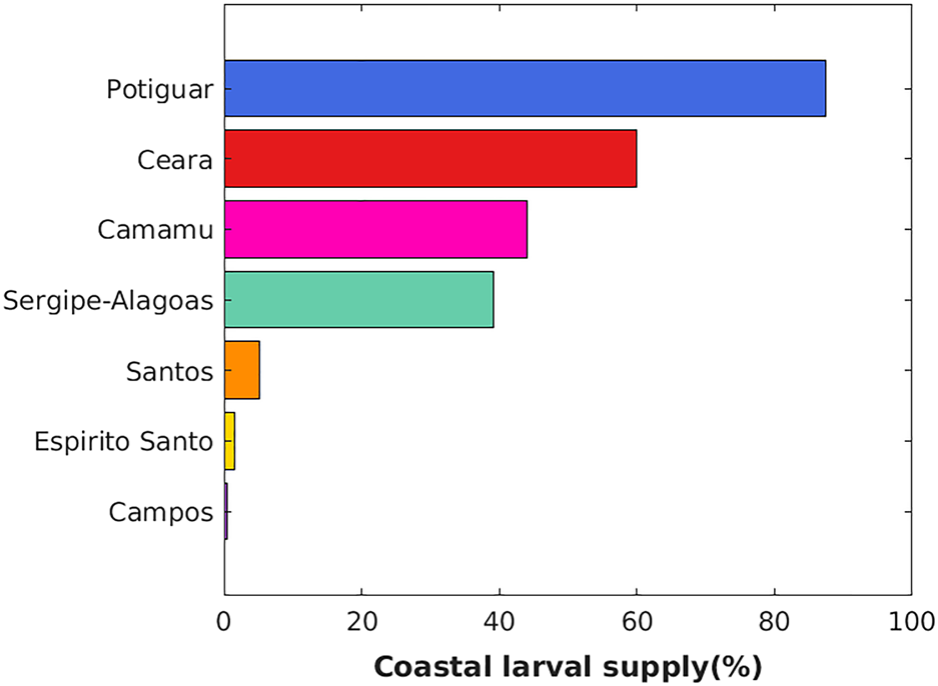
Effectiveness of the Brazilian oil-producing basins in supplying the coastline with sun-coral larvae as the percentage of total living particles released in each basin reaching the coast computed from all simulations. Created with Matlab R2018a (http://www.mathworks.com).

Our results indicate the position of oil platforms relative to regional surface flow as a crucial factor for their importance as vectors for sun-coral dispersion on the Brazilian coast. A total of 12 coastal zones were defined as receiving areas or destinations for larvae released from oil platforms. We show in Fig. 6 that the southern basin of Santos with eight release areas impacts only three receiving areas (Campos to the north, Santos itself and Pelotas to the south). In contrast, Camamu with only one release site (and one oil platform) provides larvae for nine other basins (including its own). Here, the strong seasonality of larval supply is also clear with the Sergipe-Alagoas and Camamu platforms providing larvae to all other basins but the southernmost Pelotas (PEL) (total of 11/12 receiving areas) during the winter and only five during the summer. The northern Amazonas (AM), Pará-Maranhão (PAMA), BAR (Barreirinhas), Ceará (CE), and Potiguar (POT) receiving areas did not have any larvae from the Sergipe-Alagoas Basin during the summer, and Pernambuco-Paraíba (PEPB) received larvae only during the winter. The most connected basins are Campos (CAM) and Santos (SAN), which received larvae from as far north as the Sergipe-Alagoas basin during both seasons. Despite being located at the southernmost region of the Brazilian coast, the receiving area PEL is supplied with larvae originating mainly from the Campos and Santos basins. The effectiveness of each oil-producing basin as vectors for the entire Brazilian coastline, measured as the percentage of larval supply, highlights the importance of the northern Ceará (59.89%) and Potiguar (87.47%) basins and the more central Camamu (44.11%) and Sergipe-Alagoas (39.20%) basins (Fig. 7). Only a small percentage (less than 6%) of larvae originating from the Espírito Santos, Campos, and Santos basins reached some portion of the coastline at any time.

**Figure 8:**
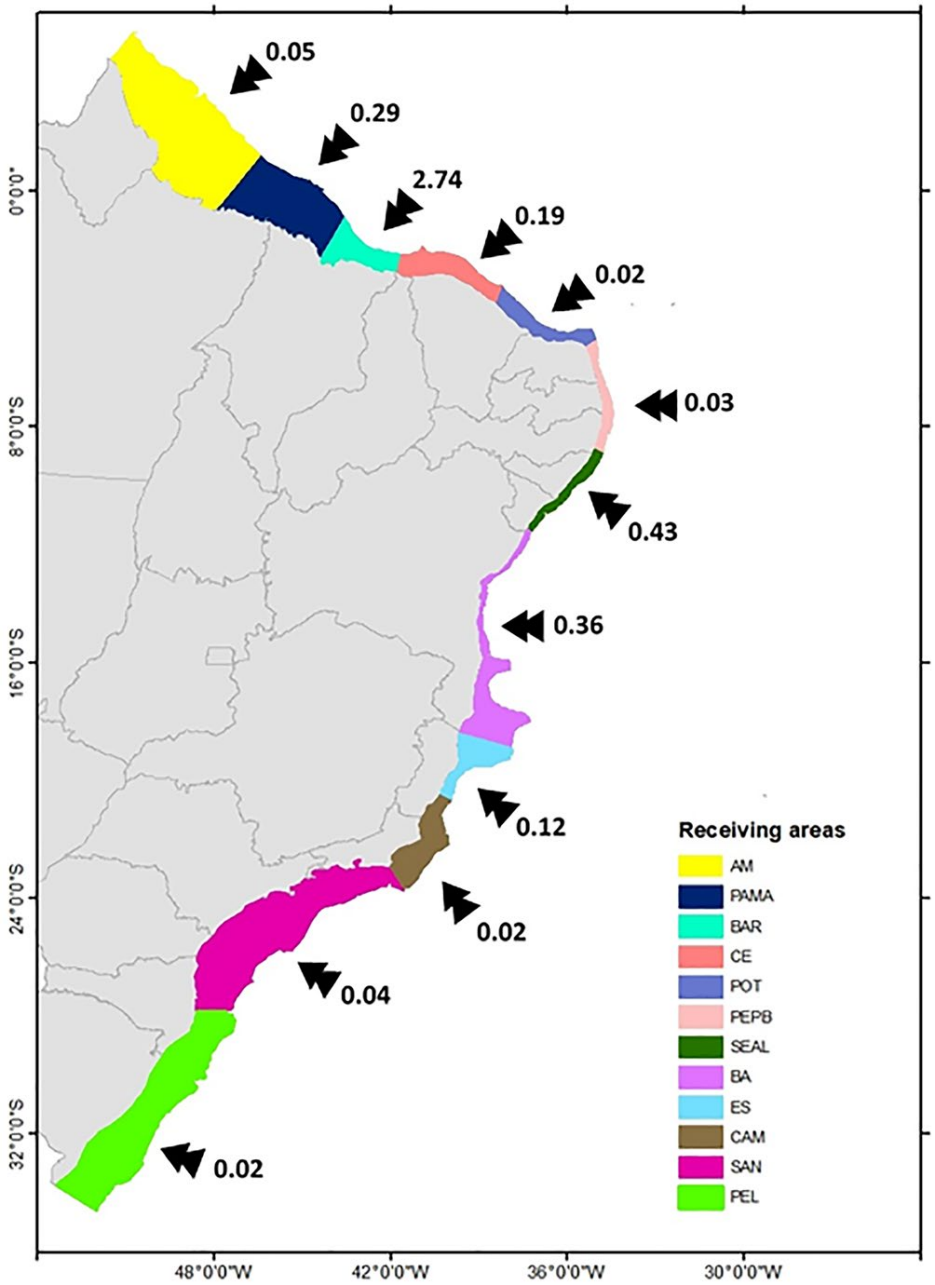
Total number of larvae per km^2^ that reach each receiving area during all the experiments. Color code for the destination basins is in the insert and abbreviations are the same as in Fig. 6. Created with ArcGIS 10.6.1 (https://www.esri.com/).

Taking the receiving areas as a whole, there are no significant interannual differences of the total number of living larvae arriving at the coast, neither for summer (Kruskal–Wallis test, *p* = 0.924) nor winter (Kruskal–Wallis test, *p* = 0.5478) (Supplementary Fig. S8). Moreover, no seasonal differences in the total number of living larvae arriving at the receiving areas were found (Kruskal–Wallis test, *p* = 0.3263) (Supplementary Fig. S9). To produce a balanced comparison among receiving areas, we computed the incidence of incoming larvae by correcting for differences in continental shelf area (Fig. 8). We found differences of one order of magnitude (relative to total number of released larvae) in the number of larvae reaching the northern Barreirinhas (BAR, 2.74 larvae/km^2^) basin compared to the next most common destinations like Sergipe-Alagoas (SEAL, 0.43 larvae/km^2^) and Bahia (BA, 0.36 larvae/km^2^). In fact, there is a significant difference between the number of larvae/km^2^ arriving the receiving areas (Supplementary Fig. S10) both for summer (Kruskal–Wallis test, *p* = 6.730 × 10^–08^) and winter (Kruskal–Wallis test, *p* = 6.019 × 10^–07^), considering the 6 years of simulation. It is worth noting that despite the Campos oil-producing basin having the largest number of oil platforms (59 distributed in seven release sites, see Supplementary Table S1), its respective receiving area (CAM) receives only 0.02 larvae/km^2^ (Fig. 7).

## Discussion

Our study reveals how inshore and offshore oil platforms behave as vectors for the dispersion of sun-coral along a large latitudinal gradient, from tropical to temperate environments, under two different western boundary current regimes. Connectivity between source and receiving areas is generally limited by the ability of invasive species to withstand environmental conditions, namely temperature^43^, their PLD and the intensity and direction of surface flow. Sun-corals have large temperature tolerance and relatively long PLD making them formidable invaders to a range of habitats from tropical to temperate. Not surprisingly, the low lethal minimum temperature for these species (≤ 12.5 °C^42^) and a yet unknown maximum lethal temperature enabled the vast majority of larvae to remain alive during the 90 days of simulations. In fact, the occurrence of *Tubastraea* spp. from warm tropical to cool temperate waters, attests their wide thermal tolerance, a common feature of successful invaders^21,44–47^. Besides temperature tolerance, *Tubastraea*'s maximum PLD of 90-days resulted in long distances traveled by sun-coral larvae in our simulations, reaching up to 7000 km (for larvae released from Sergipe-Alagoas Basin in Fig. 2). Average distances ranged from 1000 to 3000 km, well beyond the maximum distance covered by larvae from other marine organisms^48^, which vary from a few meters to hundreds of kilometers^33,49^. In general, the distance traveled by larvae is positively correlated to the PLD, so that the longer a larva stays in the plankton, the greater the distance it will likely disperse^49^. A long-lasting larval stage is certainly an important factor for the persistence of sun-corals^50^ but we have shown that under complex oceanographic settings, the location of oil platforms is a key determinant factor in the their dispersal for coastal habitats.

Under the above simulation conditions (temperature, PLD and currents) we found that larval dispersion from oil platforms is mostly controlled by the surface flow of the western boundary currents NBC and BC. The direction and seasonal variability of these currents play an important role in the way oil platforms could act as stepping-stones to the invasion of coastal habitats. The NBC flows along the continental slope (centered at 500 m depth^35^) influencing the northern oil platforms at Ceará and Potiguar basins. These platforms are located at depths shallower than 200 m and capture the weak summertime westward currents over the shelf. Larvae respond to this regime by traveling shorter distances (Fig. 2) and remaining longer in the inner shelf during the austral summer (Fig. 5). Wintertime larval transport is influenced by the onset of the NBC retroflection between 5° and 10° N, entering the NECC, causing larvae to travel longer distances compared to summer. Further to the south, the direction of larval dispersal from oil platforms at Sergipe-Alagoas and Camamu basins is more variable and occurs in opposite directions. Larvae can be predominantly advected to the south in summer or to the north and south in winter (Fig. 3), with some interannual variability (see Supplementary Figs. S1 and S2). This change in trajectories coincides with the seasonal meridional shift in the position of the bifurcation of the southern branch of the South Equatorial Current (SECs), roughly bounded between 10° and 14°S near the surface^51^. The main driver of this seasonality is the meridional shift of the Intertropical Convergence Zone (ITCZ) that moves southwards in the (austral) summer and displaces the bifurcation of the SECs northwards (maximum in November) when larvae tend to be captured by the BC southward flow. When the ITCZ shifts to the north in winter, the bifurcation of the SECs reaches its southernmost position (maximum in July) causing some larvae from Sergipe-Alagoas and Camamu basins to be also advected northwards by the weaker NBSC that feeds the NBC northwestward flow. At this time of the year, the NBC is intensified^35^, which enhances the potential of dispersion (Fig. 3) by allowing larvae to enter the NBC retroflection and the eastward NECC flow. Also note that, at the end of the wintertime simulations, most of the particles over the north and northeast Brazilian shelf have originated from further south at the Sergipe-Alagoas basin (Fig. 3).

Longer distances traveled by larvae released from Ceará and Potiguar basins during the winter lead to high rates of mortality by advection (Fig. 4). These can be attributed to the proximity of its source areas to the northwest limit of the simulation domain. These larvae are primarily dispersed by the NBC flow and, therefore, the intensity of this western boundary current during the winter may have favored the transport of a larger proportion of larvae outside the simulation domain. However, it is important to consider that the dispersion may be constrained only partially, as sun-coral larvae were also transported eastwards by the NECC. Pushing the open boundary of the model domain towards the west would possibly have allowed some larvae from northern Brazilian oil platforms to be transported by Guyana and Caribbean currents into the Caribbean Sea. This hypothesis represents an environmental threat that should be tested in future work, as it could intensify the biological invasion already existing in that area^52^. Also, model bias and RMSD of surface currents tend to be slightly larger near the equator, especially for the zonal component (Supplementary Figure S6). Negative bias of the zonal flow close to the north Brazilian coast suggests that modeled westward larval transport could be stronger than expected.

From the total 26 release sites used in our simulations, 18 sites (69%, see Supplementary Table 2) are at the southern Espírito Santo, Campos and Santos basins, mostly located close to the BC core that flows around 2000 m depth (Fig. 1). The region is characterized by the presence of quasi-steady Lagrangian transport structures driven by semi-permanent eddies and meanders^53^. These mesoscale features originate from the combination of the abrupt change in the coastline orientation, the bathymetric gradient, and also by the baroclinically unstable nature of the BC^54,55^. Final positions and larval density shown in Figs. 3 and 5, respectively, provide clear indications of how this eddy-dominated flow traps most larvae offshore where they travel long distances without actually reaching far-away coastal habitats (see Fig. 6). The persistence of these eddies along the year results in particles becoming trapped in the southern region during both austral summer and winter. This is also confirmed by similar dispersal patterns of larvae released from Campos and Santos basins (Supplementary Fig. S4). Therefore, the BC seasonality is also not so evident in the distance traveled by all larvae with average values ranging between 1000 and 2000 km (see Fig. 2). In fact, the Lagrangian transport patterns in this region described by Gouveia et al.^53^ produce a cross-shelf transport barrier to floating particles. However, there is one place where particles can reach the coast south of 24° S. Our simulations also show an important zonal transport of sun-coral larvae towards the coast that coincides with this latitude in Fig. 5.

Large scale in situ assessments of larval dispersion and regional flow depend on expensive logistics and involve measuring physical parameters and collecting a large number of samples over a wide area. However, good quality biophysical simulations can shed some light on important processes governing the connectivity of oil platforms with the coast. This is exemplified in our connectivity analysis, shown in Fig. 6, where a small fraction of all source areas, like those in the Sergipe-Alagoas and Camamu basins, can potentially contribute to the spread of larvae along nearly all the Brazilian coast (except for the southernmost Pelotas basin). However, the reason why some source areas contribute more than others possibly lies in two different regional processes. The wide dispersion range (11 receiving basins out of 12) of four release sites (sites 5 to 8 in Fig. 6) results from the seasonal north–south excursion of larvae. This excursion is coincident with changes in the mean position of the bifurcation of the SECs at the surface^38,39,56^ associated with local ITCZ-driven changes in the wind stress curl^51^. On the other hand, larvae released from 18 sites between Espirito Santo to Santos basins (sites 9 to 26 in Fig. 6) reach only five (out of 12) receiving areas because they circulate in offshore by semi-permanent eddies and are unable to cross the transport barrier positioned in 200 m depth^53^. Therefore, oil platforms located close to the coast (Ceará and Potiguar) and under the influence of the bifurcation of the SECs (Sergipe-Alagoas and Camamu) can supply the coast with more larvae than those offshore influenced by the BC (Fig. 7).

The susceptibility of coastal regions to *Tubastraea* spp. invasion does not vary significantly from year to year (Supplementary Fig. S8), even with larvae trajectories not being exactly the same in either seasons, along the 6-years of simulation. In fact, this lack of significant interannual variability in our simulations is probably the result of a short simulation time (< 10 years^57^). Further work, checking the existence of decadal variability in the number of larvae arriving at the receiving areas could emphasize the need for management action on the coast. However, the susceptibility to receiving sun-coral larvae from oil platforms vary significantly among receiving areas (Supplementary Fig. S10). The most vulnerable sites are located on the north and northeast coasts (Fig. 8). These are primarily impacted by dispersal from Ceará (59.89%), Potiguar (87.47%), Camamu (44.11%) and Sergipe-Alagoas (39.20%) basins that are the most effective in providing larvae to the coast (Fig. 7). The relatively small Barreirinhas (BAR) to the north receives the largest number with 2.74% larvae per km^2^ originating from eight release sites and as far south as Camamu Basin (site 8, Fig. 7). This is also a region characterized by a semidiurnal macrotidal regime with amplitudes of up to 6 m that may contribute to the retention of larvae close to the coast, as suggested by simulations with real conditions^58^. The next most impacted region is the Sergipe-Alagoas (SEAL) receiving area with 0.43 larvae per km^2^, with a contribution that is limited to local oil platforms and from the nearby Camamu Basin (site 8, Fig. 6).

Third in the ranking of receiving areas of larvae is the Bahia littoral with 0.36 larvae per km^2^ (BA, Fig. 8). Sun-coral larval arrival in this region would seriously threaten the Abrolhos Bank, the largest and most diverse coral reef-system in the Southwestern Atlantic^59,60^. Costa et al.^61^ already pointed out that the proximity of oil platforms to this reef system could be an issue, a hypothesis confirmed herein. Furthermore, the arrival of larvae on the Bahia coast could strengthen the biological invasion process already existing in the Todos os Santos Bay, contributing to changes in local habitat complexity^13^. According to D'Agostini et al.^38^, the region is also under the influence of the surface divergent flow between NBSC and the BC, which is evident in Figs. 3 and 6. The arrival of sun-coral larvae in the northern coast of Brazil (receiving areas of PAMA and AM in Fig. 8) can be a threat to the unique 1000 km long mesophotic carbonate reef system between French Guiana and Brazil^62,63^, which include the Parcel Manuel Luís Marine State Park containing reef formations mainly composed of calcareous algae^64^. In addition to the competition with the anthozoan *Palythoa caribaeorum*^65^, the arrival of sun-coral larvae in the region can affect the survival of Brazilian endemic coral species such as *Siderastrea stellata, Favia gravida*, and *Mussismilia hispida*^66^. Successful colonization of this system may intensify the sun-coral invasion process of the Caribbean Sea due to the ecological connectivity of this region with the Brazilian biogeographical province^67,68^.

Comparatively, fewer larvae make it to the coast in Campos and Santos basins (0.02 and 0.04 larvae per km^2^, respectively) despite having nearly half of all oil platforms (see Supplementary Table S2). Persistent cyclonic and anticyclonic structures between 20° and 30°S contribute to keep larvae away from the coast, captured by quasi-stable climatological Lagrangian transport^53^, as already discussed above. In the Cabo Frio upwelling region (23°S), offshore wind-driven Ekman transport^69^ is stronger during the summer, forced by persistent northeast winds, and may also limit onshore larval transport^70,71^. Although the southeastern coast of Brazil receives a small proportion of sun-coral larvae by oil platforms, the arrival of new invaders may facilitate the invasion of other exotic species^72,73^ and intensify the several environmental impacts already observed in the region^10,20,22,74^ signaling the possibility of expanding their geographical limit further south. Moreover, the great ability of sun-coral to colonize artificial structures^27,75^, put different port structures along the Brazilian coast at risk, pointing out an urgent need to establish different monitoring protocols for the early detection of the invader.

The massive presence of sun-coral larvae in oil platforms, their long PLD and large thermal tolerance indicate that these structures provide an important substrate for sun-corals settlement, development, and dispersion^26^. It is important to bear in mind that sun-coral larvae are competent to settle from the first day that they are released from parent colonies^76^ and may re-enter the water column multiple times^77^. They can also colonize substrates up to 110 m deep as is the case of *T. coccinea*^78^. Oil platforms can act as stepping stones for these corals between their remote origin and the Brazilian coast, facilitating the geographical expansion of *Tubastraea* spp. in the Southwest Atlantic^79^. Besides, different types of vessels, including oil tankers^8,17^, and marine litter around the kernels are subject to biofouling, and so transport the non-indigenous species for other regions^21,80,81^.

All oil-producing Brazilian basins can act as a source of sun-coral larvae to the coast, with both the proximity of the platforms to the coast and the seasonal variability of surface flow being important factors determining the potential impact of an oil-producing region on the spread of sun-coral. We hope that by identifying those oil-producing regions with the greatest potential to supply the Brazilian coast with larvae (Fig. 7), the present study can contribute to the decommissioning planning of oil units and the installation of new structures regarding their potential for enhancing biological invasion^82^. This information supports the requirement of environmental counterparts for oil exploration in the region, and the urgent need to establish antifouling protocols on oil platforms, which have been absent in Brazil and in the world so far^83^. Each new invasion, or intensification of pre-existing invasion, brings unpredictable changes to the marine environment that need to be addressed. The identification of areas susceptible to the arrival of sun-coral larvae has the potential to reduce the widespread impact, acting as a critical tool for maintaining the ecological integrity of the habitats^84^, and minimizing the costs of control and mitigating actions^85^. Thus, with the biological assumptions adopted, the biophysical modeling presented here is an important first step towards understanding the patterns of natural dispersion of sun-corals in the Southwest Atlantic. The application of biophysical modeling in different spatial and temporal scales, can provide a basis for several studies focused on the dispersion and impacts of the sun-coral around the world.

## Data and methods

In silico experiments of larval dispersion of sun-coral were conducted to determine the role of oil platforms as vectors for coastal supply. Biophysical experiments started with the ocean simulation using the Regional Ocean Modeling System (ROMS^86,87^, Rutgers version, available at https://www.myroms.org/), that generated the three-dimensional ocean fields used to run the biological individual-based model Ichthyop^88^ (version 3.3.6, available at https://www.ichthyop.org/).

### Study area

The study region is in the Southwest Atlantic Ocean and contains all the Brazilian oil-producing marine basins: Ceará, Potiguar, Sergipe-Alagoas, Camamu, Campos and Santos. The oil platforms of these basins, used as sun-coral release areas, are located in different flow regimes and depths, with the northern platforms in shallow waters and southern offshore platforms (Fig. 1). The twelve sites corresponding to the potential receiving areas are located along the Brazilian coast (Fig. 8) and have different sizes (see Supplementary Table S1).

### Hydrodynamical model

The ROMS was applied to generate the physical processes in the domain bounded at 6° N–36° S and 24° W–53° W, with a horizontal resolution of 1/12° (approximately 9.3 km) and 30 sigma levels. This model resolution has already indicated the ability to solve mesoscale features in the study area^38,39,56,89^. The model was forced at the surface using 6-hourly atmospheric fields derived from the Climate Forecast System Reanalysis (CFSR^90^), available at https://rda.ucar.edu/datasets/. The open boundaries of the domain (north, south, and east) were forced by 5-day ocean fields of temperature (in °C), salinity (in PSU), surface currents (in m/s), and sea surface height (m) obtained from the Simple Ocean Data Assimilation reanalysis (SODA^91^, version 3.3.1, available at https://www.atmos.umd.edu/~ocean/). A Flather-type condition^92^ was applied for the barotropic mode, a Chapman-implicit condition^93^ for the free surface, a Clamped condition for baroclinic mode, and the combination of radiation and nudging^94^ were used for the inertial passive tracers. The simulation also included the freshwater discharge from the Amazonas, Doce, São Francisco, and Paraíba do Sul rivers (estimated by the Brazilian National Water Agency, available at http://www.snirh.gov.br/hidroweb/), and tidal constituents extracted from the TPXO global tidal model, version 8, available at https://www.tpxo.net/global/tpxo8-atlas^95^.

The free-run simulation was performed from 2004 to 2015, discarding the first 2 years as spin up^96^. Mean monthly surface fields from 2006 to 2015 were compared on a seasonal basis to the Operational Sea Surface Temperature and Sea Ice Analysis (OSTIA, available at https://resources.marine.copernicus.eu/ SST_GLO_SST_L4_REP_OBSERVATIONS_010_011) and to the Ocean Surface Current Analyses Real-time (OSCAR, available at https://podaac.jpl.nasa.gov/dataset/OSCAR_L4_OC_third-deg). The good agreement between the simulated and satellite-derived seasonal SST fields is confirmed by low values of RMSD and BIAS (Supplementary Fig. S5). The comparison statistics of the zonal (*u*) and meridional (*v*) surface velocity seasonal fields also indicate that the model reproduces the main currents and their seasonal variability (Supplementary Figs. S6, S7). Therefore, our simulations accurately represent the main surface flow patterns and variability in the Southwest Atlantic, hence contributing to the reliability of the biophysical simulations. Moreover, the vertical gradient of sea temperature and salinity were compared with in situ profiles from the Prediction and Research Moored Array in the Tropical Atlantic moorings (PIRATA, available at https://www.pmel.noaa.gov/gtmba/), collected during the modeling period. Hourly outputs of 3D temperature, salinity, and currents fields from 2010 to 2015 were stored for austral summer (January–February–March) and winter (July–August–September) to feed the biophysical simulations.

### Biophysical simulation

The sun-coral larval dispersion was simulated by the individual based model (IBM) Ichthyop, employing the biological characteristics of sun-corals and the hourly outputs from ROMS. Biophysical experiments were conducted within the same domain of ROMS simulations, for austral summer and winter between 2010 and 2015, as sun-coral larval release occurs at any time during the year but tends to peak during these seasons^76^. Also, the 90-days duration of each experiment corresponds to the measured PLD of the genus^18^. In fact, although Luz and collaborators^18^ suggested this PLD, they also stated that *Tubastraea coccinea* larvae were healthy at the end of experiment and, therefore, may remain longer in the water-column. In order to define the number of larvae to be released, a test for differences in mortality resulting from the total number of released particles ranging from 70,000 to 200,000 were applied, and it showed no significant differences for both summer (Kruskal–Wallis test, *p* = 0.999) and winter (*p* = 0.998). Thus, to keep computation costs at a reasonable level we decided for the total release of 70,000 larvae for each one of the 12 Lagrangian experiments. The total amount of larvae released at each site was proportional to their respective sizes (Fig. 1). The general configuration used for the Biophysical Simulations is detailed in Table 1.

The source areas of sun-coral larvae were based on the location of the 136 oil platforms in the Brazilian oil-producing marine basins during the period, according to the National Oil Agency (ANP, available at http://www.gov.br/anp/pt-br) and Brazilian Navy (available at http://www.marinha.mil.br). To improve comprehension of the results and reduce computational costs these platforms were grouped based on proximity among them. A 9 km-buffer for each platform was created in the ArcGIS (version 10.6.1), respecting the spatial resolution of ROMS outputs. Nearby buffers overlapped generating bigger areas. Thereby, 26 Source Areas were established at the domain (Fig. S1).

### Mortality, spatial distribution, and dispersal patterns

The Ichthyop outputs were used to estimate the advection mortality rates (MA), the spatial distribution, and the dispersal patterns of sun-coral larvae. The MA was calculated for each source area by dividing the sum of the larvae released by the area that was advected outside of the domain and the total larvae released by this area (Eq. 1). The Mann–Whitney non-parametric test was used to verify the existence of significant differences between the mortality rates between austral summer and winter. To assess the interannual difference in larval mortality and the difference in mortality among release zones, the Kruskal–Wallis test was applied, which is an extension of the previous test, both with a 5% significance.

1 $$MA=\frac{\sum {a}_{sa}}{\sum {d}_{sa}}$$

where ${a}_{sa}$ is the number of larvae advected outside the domain from each source area, ${d}_{sa}$ is the number of larvae released from each source area.

The spatial distribution of the larvae was evaluated through the application of the Kernel Density Estimator in the positions of the larvae during the entire simulation period^98^. This method indicated the likelihood of larvae to be found in each cell of the domain, assembling all years of austral summer and winter.

The dispersion patterns of the sun-coral larvae were analyzed based on the distance traveled by the living particles and the destination reached by them at the end of the simulations. The total distance traveled, in kilometers, by surviving larvae was calculated using the sw_dist function of the Matlab software R2018a (http://www.mathworks.com). The destination of the particles refers to the position of the alive larvae on the last day of each simulation inside the domain. With this last information, it was possible to assess the contribution of each Source Area (SA) in sun-coral larval supply to the 12 Receiving Areas (RA) on the Brazilian coast, defined by the Brazilian basin’s limits and the 200 m isobath. Thus, the Larval Supply (LS) rate of each release zone to each receiving zone was calculated (Eq. 2), and the results displayed into matrices for austral summer and winter.

2 $$LSSA,RA \left(\%\right)=\frac{\sum \; larvae \; released \; from \; SA \; that \; reached \; RA }{\sum \; larvae\; that \; reached \; the \; RA\; (independent \; of \; the \; source)} \times 100$$

where SA is the source area and RA the receiving area.

The total number of larvae released in each experiment (70,000) is likely not realistic, precluding an assessment of the strength of connectivity. Therefore, these should be interpreted as an indication of the probability that larvae spawned from a release site (one or more platforms) successfully settle on a receiving area on the coast.

We used the Kruskal–Wallis test to assess the interannual and seasonal differences of total larvae arriving at the receiving areas as a whole, and the difference of living larvae per km^2^ arriving at receiving areas, with a 5% significance.

## Supplementary Information

Supplementary Information.
