# Supplementary material for: Western boundary currents drive sun-coral (Tubastraea spp.) coastal invasion from oil platforms
Source: Sci Rep. 2022 Mar 28;12:5286. doi: 10.1038/s41598-022-09269-8 (PMC8960833; doi:10.1038/s41598-022-09269-8)
Supplement: Supplementary file 1 — Supplementary Information. [file 41598_2022_9269_MOESM1_ESM.pdf]

Western boundary currents drive sun-coral (*Tubastraea* spp.) coastal invasion from oil platforms

Stella Correia Cesar Coelho[1], Douglas Francisco Marcolino Gherardi[1], Mainara Biazati Gouveia[2], Marcelo Visentini Kitahara[3,4,5].

[1] Laboratory of Ocean and Atmosphere Studies (LOA), Earth Observation and Geoinformatics Division, National Institute for Space Research (INPE), São José dos Campos, SP, Brazil.

[2] Earth and Environmental Physics Department (DFTMA), Physics Institute, Ondina Campus, Federal University of Bahia (UFBA), Salvador, Brazil.

[3] Institute of Marine Sciences, Federal University of São Paulo (UNIFESP), Santos, Brazil.

[4] Center for Marine Biology, University of São Paulo (USP), São Paulo, Brazil.

[5] Department of Zoology (Invertebrate Zoology), National Museum of Natural History, Smithsonian Institution, Washington, D.C., United States of America.

Supplementary information:

Receiving Area	Area (km^2)
AM	168871
PAMA	79516
BAR	26545
CE	30425
POT	20826
PEPB	13261
SEAL	11835
BA	47383
ES	23685
CAM	32054
SAN	134518
PEL	110887

Supplementary Table S1. Size (km^2) of receiving areas along the Brazilian coast.

Oil-producing basin	Source area	Oil platforms
Ceará	1	PCR-1; PCR-2; PEP-01
	2	PAT-1; PAT-2; PAT-3; PXA-1; PXA-2; PXA-3
Potiguar	3	BIQ-1; PAG01; PAG02; PAG03; PART1; PART2; PCIO-1; PUB01; PUB02; PUB03; PUB04; PUB05; PUB06; PUB07; PUB08; PUB09; PUB10; PUB11; PUB12; PUB13; PUB15; POUB-1; POUB-2
	4	PARB1; PARB3; PPE-1B; PPE-2; PPE-3
Sergipe-Alagoas	5	PCB01; PCB02; PCB03; PCB04; PCM-01; PCM02; PCM03; PCM04; PCM05; PCM06; PCM07; PCM08; PCM09; PCM10; PDO01; PDO02; PDO03; PGA-01; PGA-03; PGA-03; PGA-07; PGA-08;
	6	PRB01
	7	FPPRM
Camamu	8	PMNT-1
Espírito Santo	9	FPSOs Cidade de Vitória, Cidade São Mateus
	10	PCA-1; PCA-2; PCA-3
	11	PPER-1
Campos	12	Polvo; Peregrina A e B
	13	OSX3; Polvo A
	14	FPSOs Cidade de Niterói, Fluminense, Frade, Marlim Sul; P-07; P-08; P-09; P-12; P-15; P-18; P-19; P-20; P-25; P-26; P-31; P-33; P-35; P-37; P-40; P-43; P-47; P48; P-50; P-51; P-52; P-53; P-54; P-55; P-56; P-62; P-65; PCH-1; PCH-2; PCE-1; PGP-1; PNA-1; PNA-2; PPM-1; PPG-1; PVM-1; PVM-2; PVM-3; PCP-1; PCP-2; PRA-1
	15	FPSOs Capixaba, JK, Cidade Anchieta, Espírito Santo, P-58, P-57
	16	FPSO Cidade do Rio de Janeiro
	17	P-63
	18	OSX1
Santos	19	FPSOs Cidade de Ilhabela, Cidade de São Paulo
	20	FPSO Cidade de Angra dos Reis
	21	FPSO Cidade de Paraty
	22	FPSO Cidade de Mangaratiba
	23	FPSO Cidade de Santos
	24	PMXL-1
	25	FPSO Cidade de Itajaí
	26	PMLZ-1

Supplementary Table S2. List of oil platforms used to define the source areas located in the oil-producing sedimentary basins within the Brazilian Economic Exclusive Zone between 2010 and 2015.

Summer

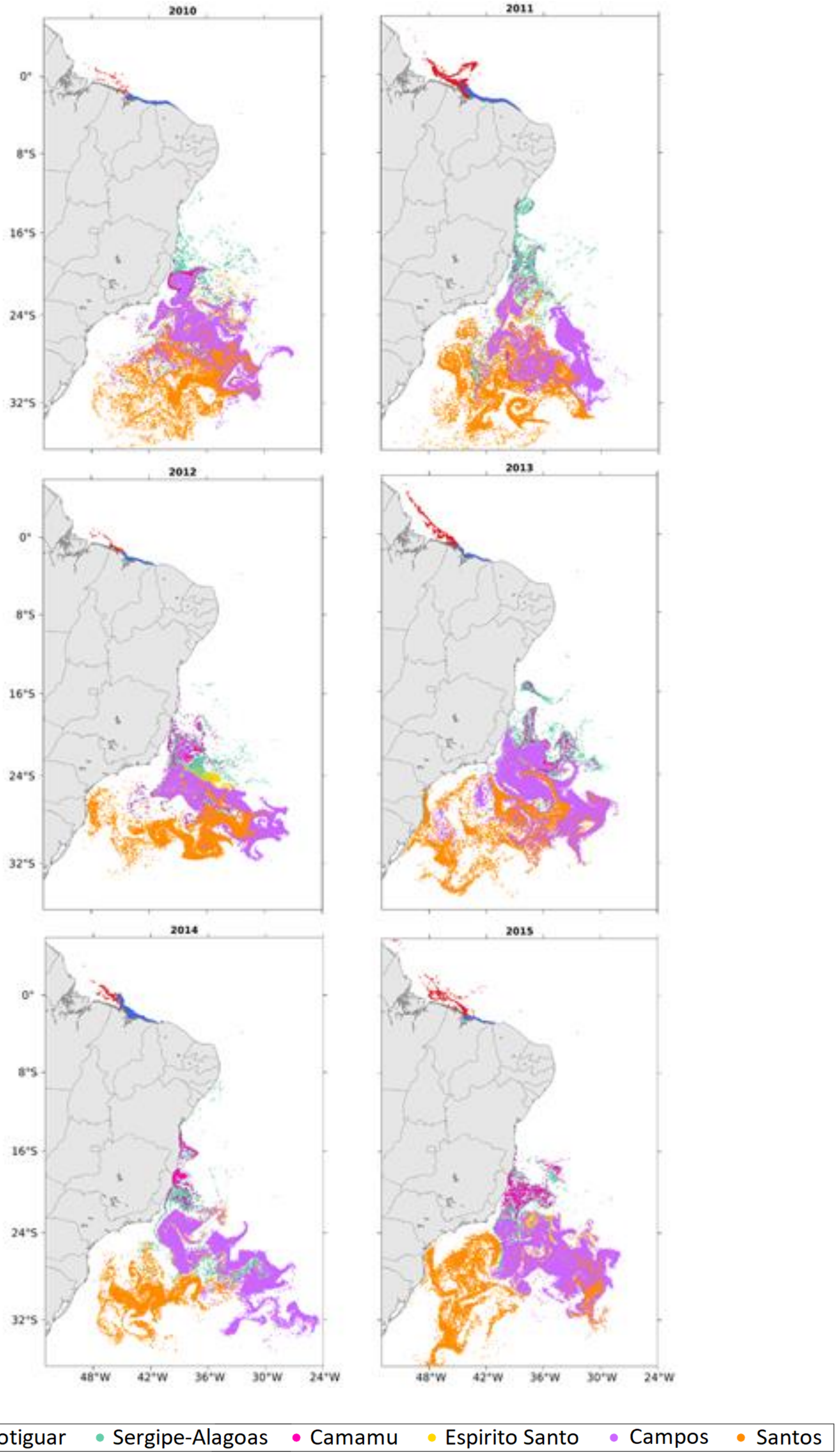

Supplementary Figure S1. Distribution of the surviving particles after 90 days of simulation during the summer between 2010-2015. Colors correspond to the oil-producing basin from where larvae were released. Created with Matlab R2018a (www.mathworks.com).

Winter

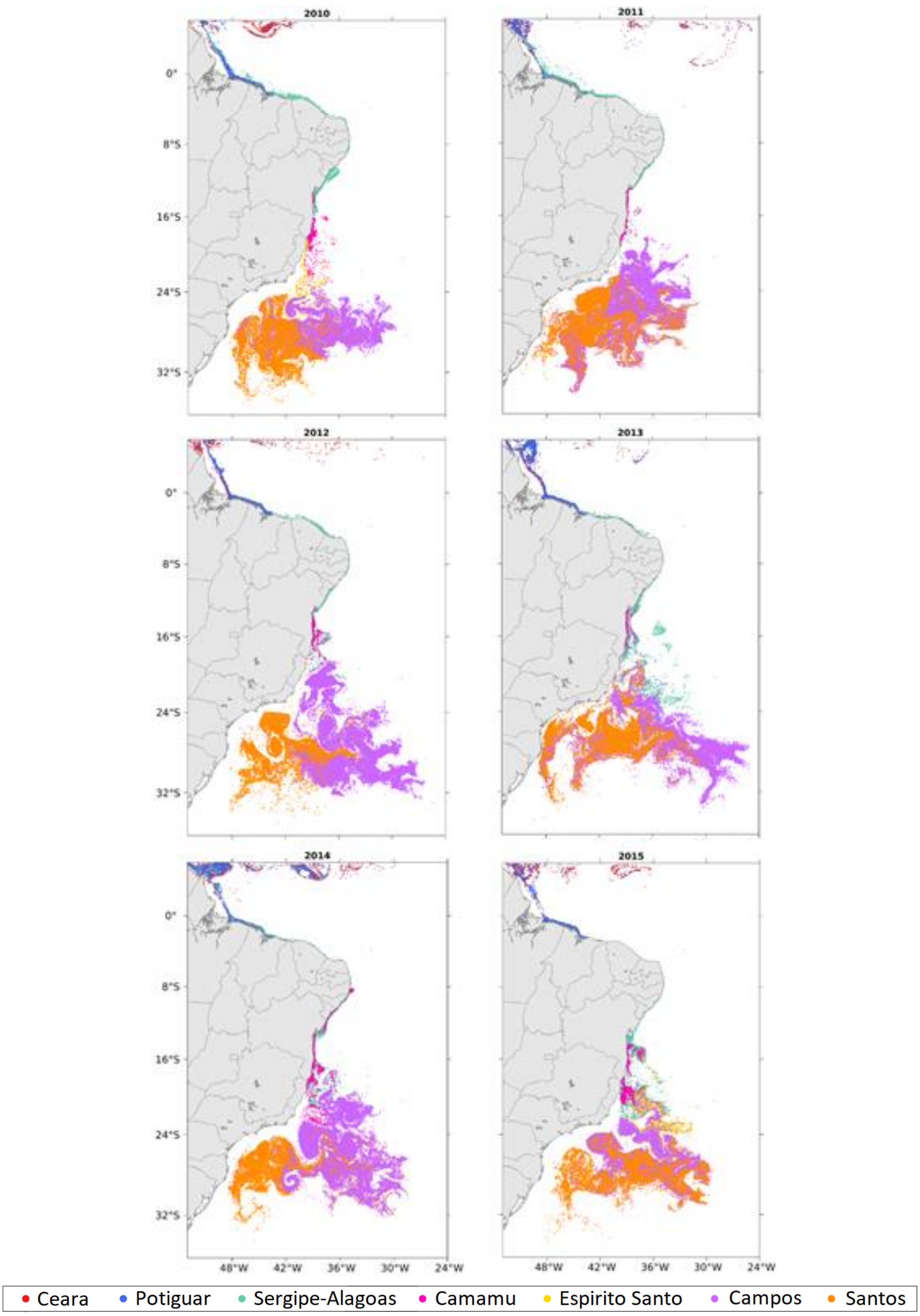

Supplementary Figure S2. Distribution of the surviving particles after 90 days of simulation during the winter between 2010-2015. Colors correspond to the oil-producing basin from where larvae were released. Created with Matlab R2018a (www.mathworks.com).

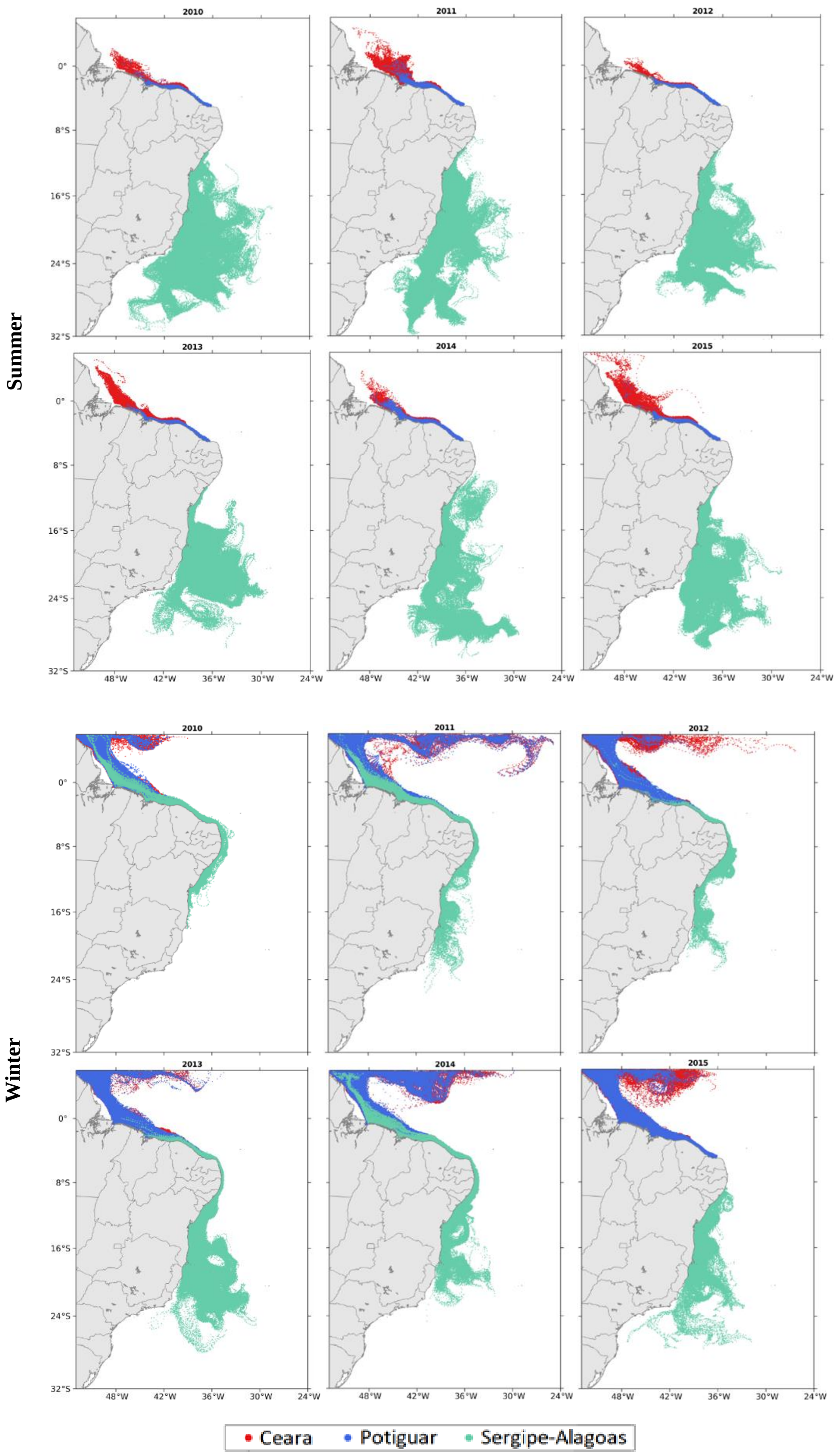

Supplementary Figure S3. Maps showing the trajectories of larvae released from Ceara, Potiguar and Sergipe-Alagoas basins along the 90 days of simulations during the summer and winter between 2010-2015. Created with Matlab R2018a (www.mathworks.com).

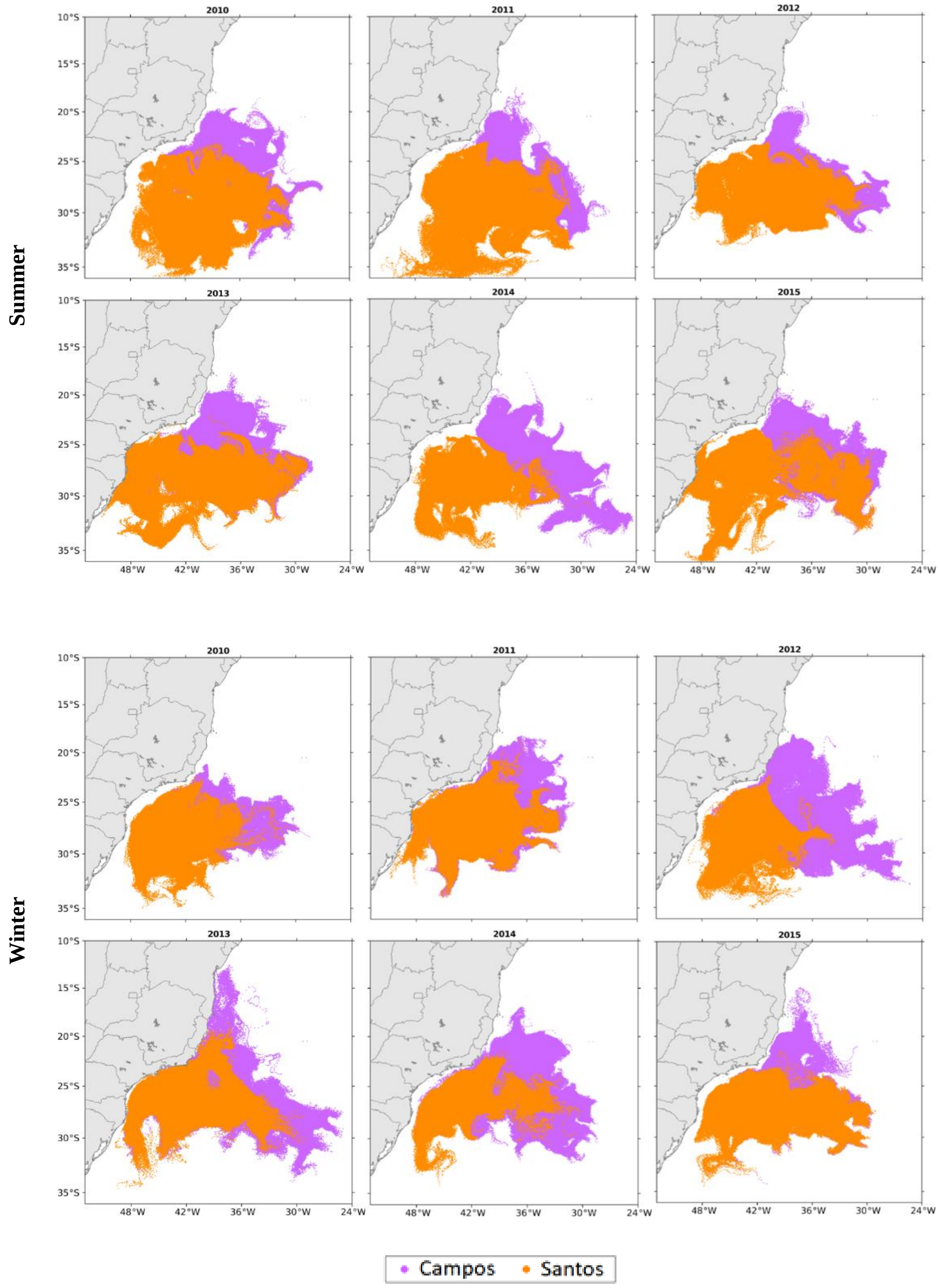

Supplementary Figure S4. Maps showing trajectories of larvae released from Campos and Santos basins along the 90 days of simulations, highlighting the influence of mesoscale structures in the retention of particles. Created with Matlab R2018a (www.mathworks.com).

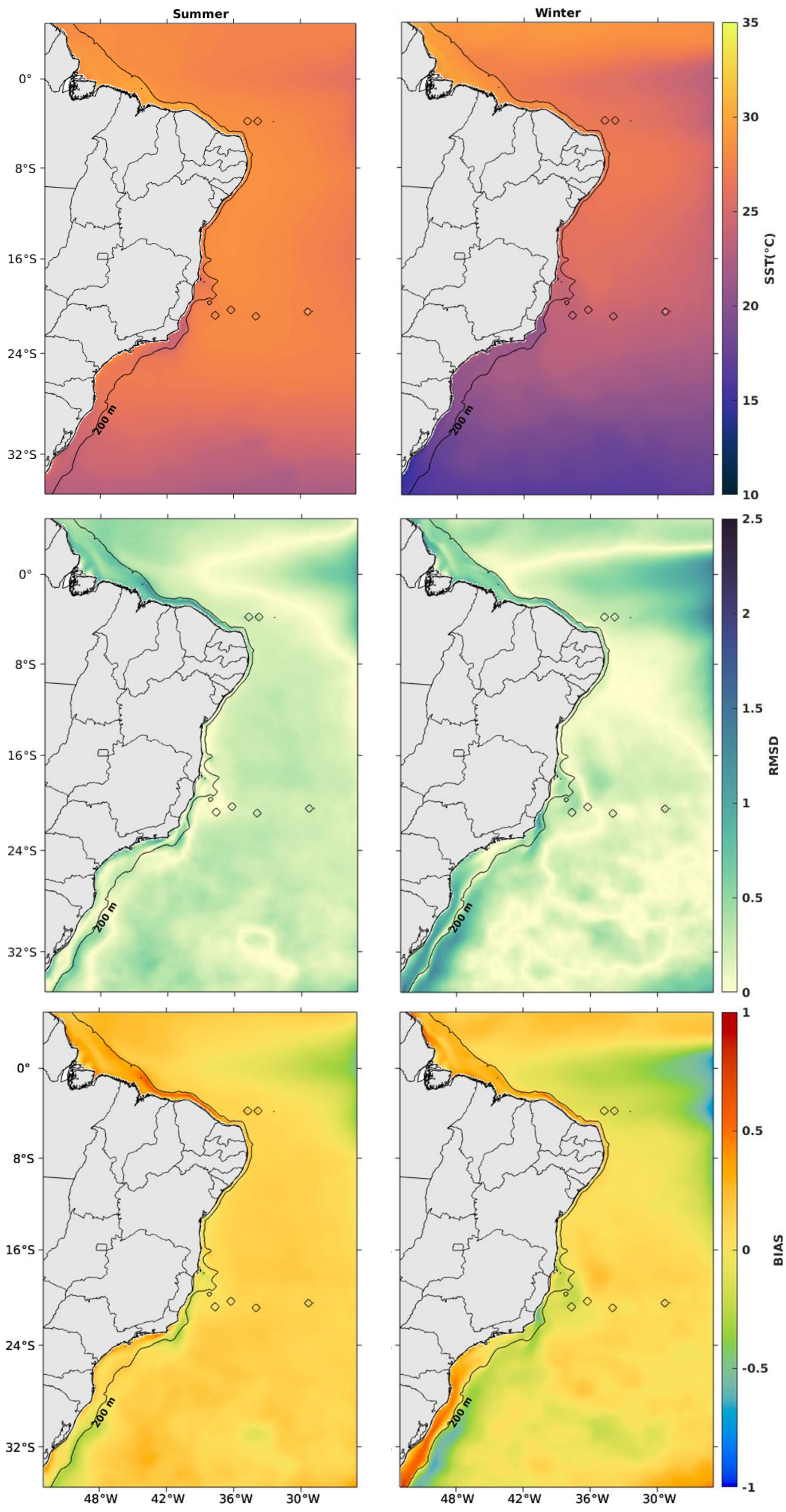

Supplementary Figure S5. Summer (left) and winter (right) model mean (2006-2015) Sea Surface Temperature (SST, °C, top panels), with root mean square deviation (RMSD, middle panels) and bias (bottom panels) relative to OSTIA. Created with Matlab R2018a (www.mathworks.com).

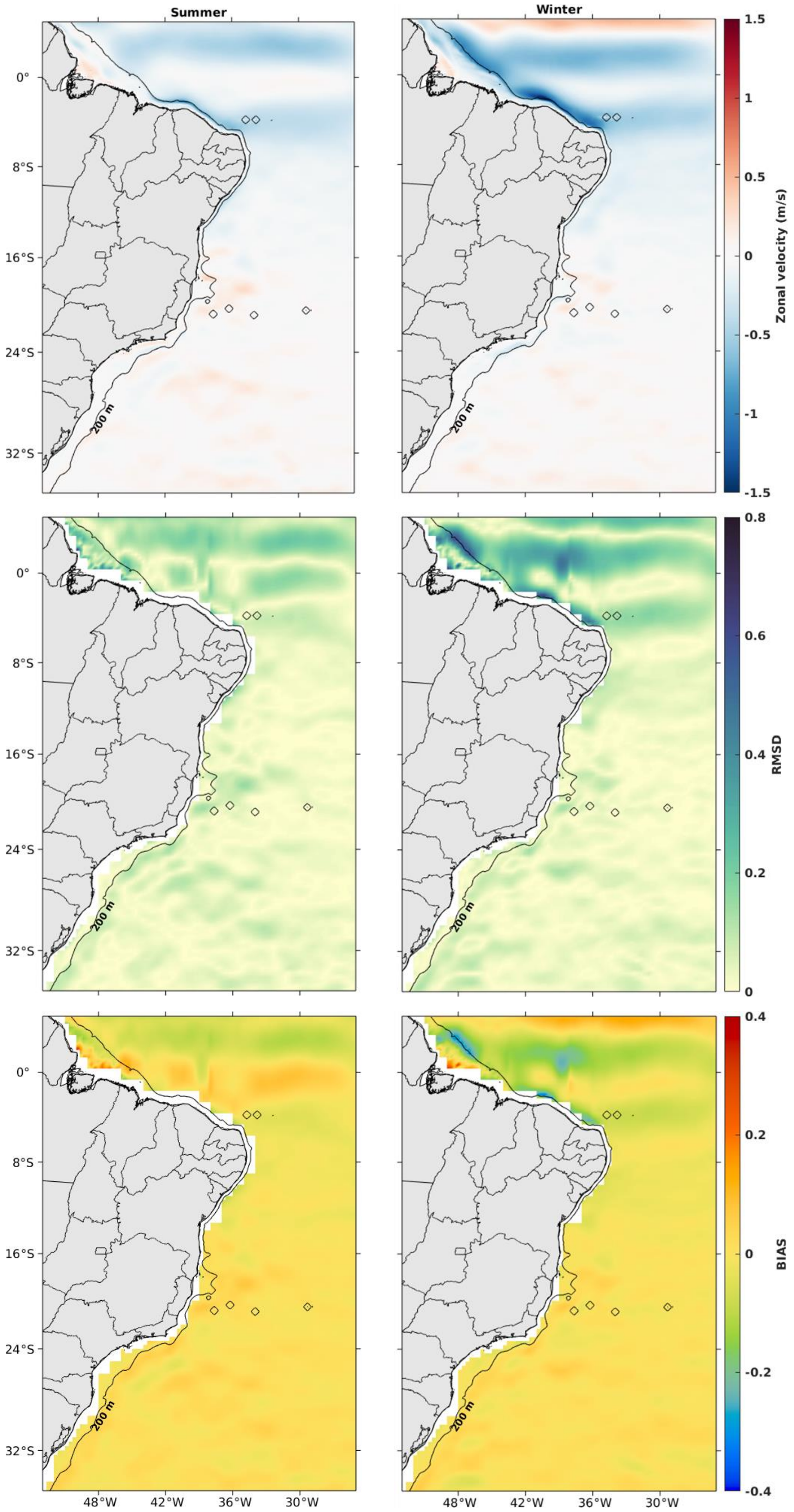

Supplementary Figure S6. Summer (left) and winter (right) model mean (2006-2015) surface velocity (m/s) of the zonal component (*u*, top panels), root mean square deviation (RMSD, middle panels) and BIAS (bottom panels) relative to OSCAR. Created with Matlab R2018a (www.mathworks.com).

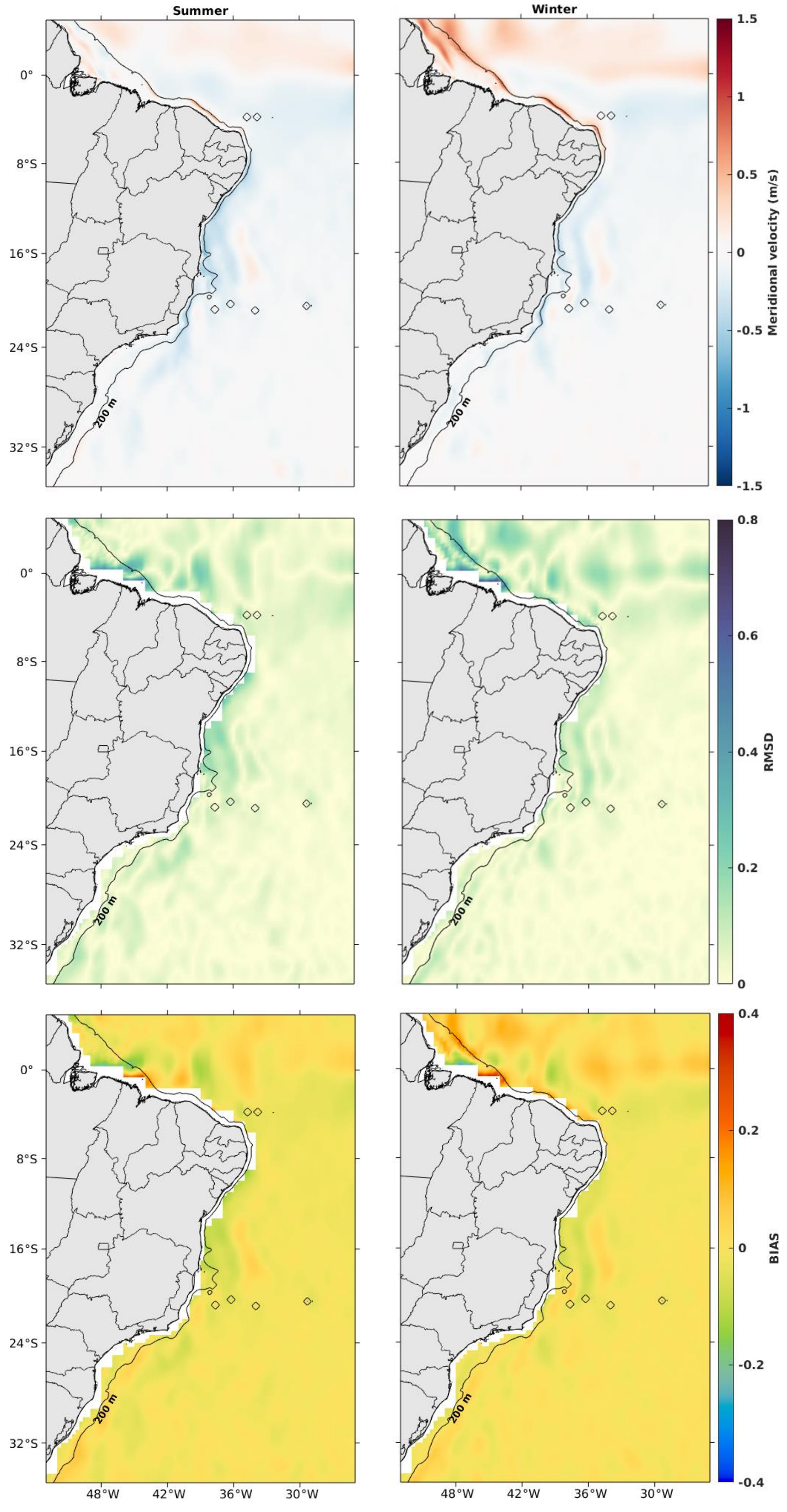

Supplementary Figure S7. Same as in Figure S6 for the meridional component (*v*) of the sea surface currents. Created with Matlab R2018a (www.mathworks.com).

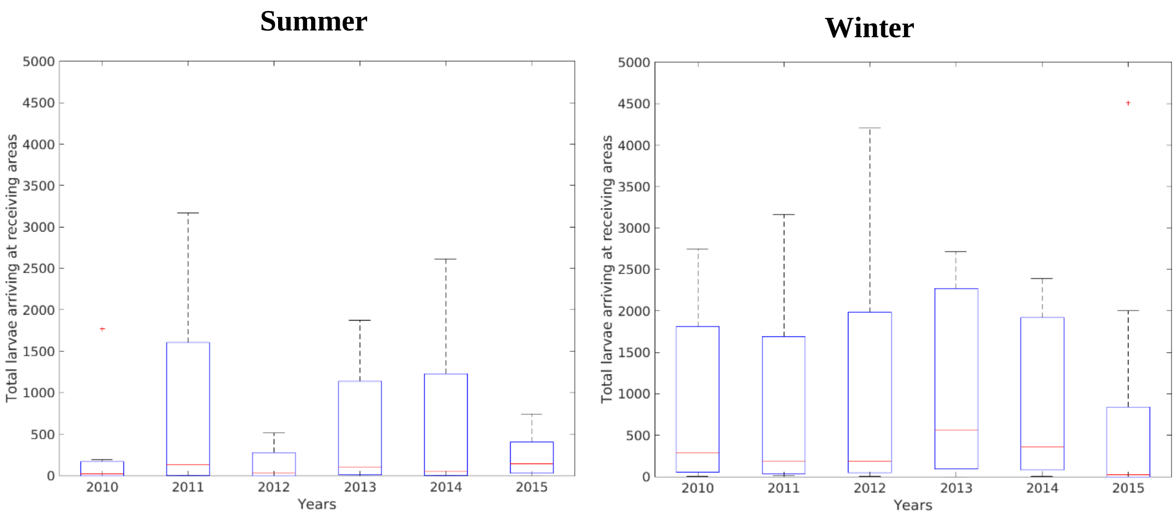

Supplementary Figure S8. Total larvae arriving at receiving areas for each year during winter and summer. In the boxplots, the central mark indicates the median, the whiskers indicate extreme data points and red crosses are outliers. Created with Matlab R2018a (www.mathworks.com).

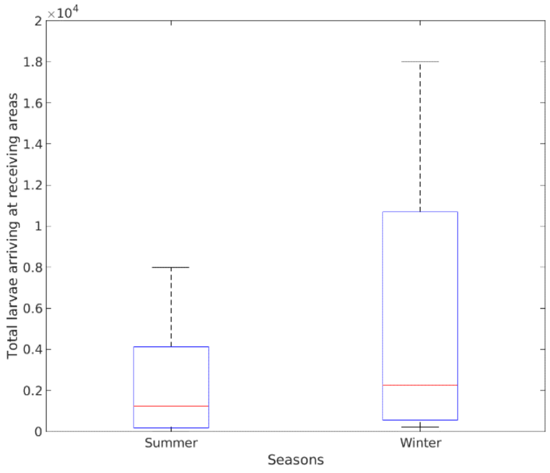

Supplementary Figure S9. Seasonal differences of total larvae arriving at receiving areas for all years. In the boxplots, the central mark indicates the median, the whiskers indicate extreme data points and red crosses are outliers. Created with Matlab R2018a (www.mathworks.com).

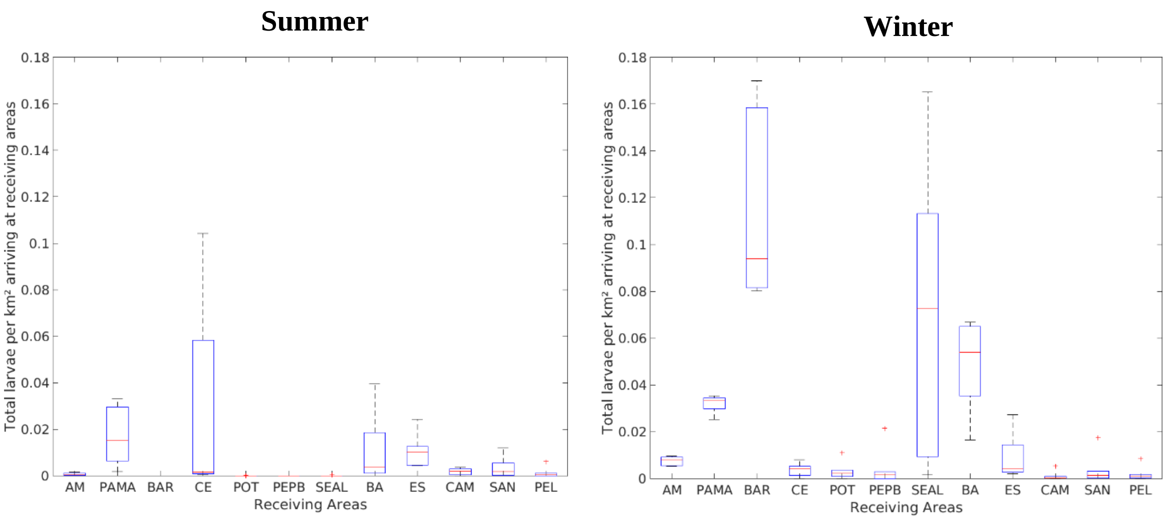

Supplementary Figure S10. Total (2010-2015) living larvae per km^2 arriving at receiving areas, during the winter and summer. Central mark indicates the median, whiskers indicate extreme data points and red crosses are outliers. Created with Matlab R2018a (www.mathworks.com).
